# Impaired Antitumor Immune Response in *MYCN*-amplified Neuroblastoma Is Associated with Lack of CCL2 Secretion and Poor Dendritic Cell Recruitment

**DOI:** 10.1158/2767-9764.CRC-21-0134

**Published:** 2022-07-05

**Authors:** Jamila Kacher, Olivier Manches, Caroline Aspord, Hervé Sartelet, Laurence Chaperot

**Affiliations:** 1 Institute for Advanced Biosciences, Inserm U1209, CNRS UMR5309, Université Grenoble Alpes, Grenoble, France.; 2 Etablissement Français du Sang Auvergne-Rhône-Alpes, Grenoble, France.; 3 Laboratoire de Biopathologie, CHRU de Nancy, Nancy, France.; 4 Inserm U1256, Université de Lorraine, Nancy, France.

## Abstract

In neuroblastoma, *MYCN* amplification is associated with sparse immune infiltrate and poor prognosis. Dendritic cells (DC) are crucial immune sentinels but their involvement in neuroblastoma pathogenesis is poorly understood. We observed that the migration of monocytes, myeloid and plasmacytoid DC induced by *MYCN*-nonamplified neuroblastoma supernatants was abrogated by the addition of anti-CCL2 antibodies, demonstrating the involvement of the CCR2/CCL2 axis in their recruitment by these tumors. Using public RNA sequencing and microarray datasets, we describe lower level of expression of *CCL2* in *MYCN*-amplified neuroblastoma tumors, and we propose a working model for T-cell recruitment in neuroblastoma tumors in which CCL2 produced by neuroblastoma cells initiates the recruitment of monocytes, myeloid and plasmacytoid DCs. Among these cells, the CD1c^+^ subset may recruit T cells by means of CCL19/CCL22 secretion. *In vitro*, supernatants from DCs cocultured with neuroblastoma cell lines and activated contain CCL22 and CCL19, and are chemotactic for both CD4^+^ and CD8^+^ T cells. We also looked at immunomodulation induced by neuroblastoma cell lines, and found *MYCN*-nonamplified neuroblastoma cell lines were able to create a microenvironment where DC activation is enhanced. Overall, our findings highlight a major role for CCL2/CCR2 axis in monocytes, myeloid and plasmacytoid cells recruitment toward *MYCN*-nonamplified neuroblastoma, allowing further immune cell recruitment, and show that these tumors present a microenvironment that can favor DC responses.

**Significance:**

In *MYCN*-nonamplified neuroblastoma, CCL2 produced by neuroblastoma cells induces the recruitment of antigen-presenting cells (DCs and monocytes/macrophages), allowing infiltration by T cells, in link with CCL19 and CCL22 production, hence favoring immune responses.

## Introduction

Neuroblastoma is the most common extracranial solid tumor in childhood arising from the sympathetic nervous system (1). The use of multimodal intensive treatment leads to improved survival of high-risk neuroblastoma that now approaches 50% (2). Molecular studies are mandatory in risk stratification and management planning in neuroblastic tumors. *MYCN* amplification is an important biomarker, because it is associated with advanced stages of disease, unfavorable biological features, and poor outcome. In the context of neuroblastoma, *MYCN* can modulate antigens expressed in tumor cells and thus influence immunosurveillance (3).

Immunosurveillance involves several immune cell subsets among which dendritic cells (DC) and tumor-infiltrating lymphocytes are crucial to develop antitumor effect. DC comprise a heterogeneous population of professional antigen-presenting cells (APC) that can capture, process, and present antigens to T cells, hence triggering antigen-specific immune response (4) and linking together innate and adaptive immunity. Depending on their origin, location, and function, DCs are divided into several subpopulations. Plasmacytoid DC (PDC) produce high amounts of type I IFNs during viral infections (5), they are characterized by CD123, blood DC antigens BDCA-2 (CD303) and BDCA-4 (CD304), expression while myeloid DC (MDC, also named conventional or classical DCs, cDC) express CD11c or BDCA-3 (CD141; ref. 6). MDC comprises two main subpopulations including CD1c/BDCA-1^+^ cells (cDC2) and CD141/BDCA-3^+^ (cDC1) cells that account for approximately 50% and 5%–10% of a total peripheral DC population, respectively (7). MDC are responsible for capture, processing, and presentation of antigens on their surface to T cells. A recent study demonstrated that MDC and natural killer (NK) cells are positively correlated with T-cell infiltration in human neuroblastoma, and associate with a favorable prognosis (8). Elsewhere, activated PDC can activate NK cells and are efficient at killing high-risk neuroblastoma cells (9), and because they can express TRAIL (10), they may directly kill TRAIL receptors expressing neuroblastoma cells (11).

Chemokines play a fundamental role in regulation of DC migration and function (12, 13). Chemokines are classified into four families based on the relative position of a conserved cysteine motif, namely, CC, CXC, XC, and CX3C (14). They regulate migration, adhesion, phagocytosis, cytokine secretion, proliferation, and apoptosis by activating G protein–coupled receptors (14). Interestingly, several chemokines have been found at higher concentrations in the *MYCN*-nonamplified neuroblastoma microenvironment and can be secreted by neuroblastoma cells to selectively recruit specific immune cell subsets (15). In neuroblastoma mouse models, treatments mediated by a CXCR4 antagonist augmented the efficacy of DC vaccines (16). *MYCN* amplification results in the downregulation of CCL2 in neuroblastoma cells, which seems involved in NK and T cells infiltration (17). iNKT are rare innate cells with potentially important role in antitumor immune responses and have been found enriched in neuroblastoma tumors with favorable outcome (18). They represent an actionable cell type that can be harnessed to develop innovative treatments in neuroblastoma (19). Their migratiion toward neuroblastoma cells in *MYCN*-nonamplified tumors has been shown to depend on CCL2 (20, 21). However, many mechanisms regulating immune infiltration by other immune cell subsets still remain unknown. Neuroblastoma with *MYCN* amplification are often characterized by a sparse and limited immune infiltrate in the tumor microenvironment (TME), and infiltrating immune cells lack activation markers (22). Taken together, the development of new and more effective immunotherapies is a high priority. Such development will benefit from an improved understanding of interactions between tumor cells and the immune system to increase anti-neuroblastoma cell immune responses while minimizing or blocking immunosuppressive immune responses.

Although DC are the pillars of efficient immune responses, little is known about their recruitment and function in neuroblastoma. In the current study, by using *in vitro* models, we characterized human DC subsets migration and function in the context of neuroblastoma, with a special emphasis on *MYCN*-amplified tumor. We also analyzed public genomic datasets, to study the role of the CCL2/CCR2 axis in DC recruitment in primary tumors, and its deficiency in *MYCN*-amplified tumors.

## Materials and Methods

### Cell Lines and Peripheral Blood Mononuclear Cells

Adherent neuroblastoma cell lines derived from bone marrow metastases SK-N-DZ (DZ, passage 71 to 82, RRID:CVCL_1701), SK-N-SH (SH, passage 26 to 37, RRID:CVCL_0531), and SK-N-AS (AS, passage 2 to 7, RRID:CVCL_1700) were obtained from the ATCC and IGR-N-N91 (N91, passage 32 to 42, RRID:CVCL_8883) was from Institut Gustave Roussy Paris (Villejuif, France). Their identity was verified by HLA-A and B typing (PCR sequence-specific oligonucleotide method, EFS-AURA Grenoble, HLA laboratory). The cells were tested negative for *Mycoplasma* (MycoAlert *Mycoplasma* detection kit, Lonza). The neuroblastoma cell lines were cultured in DMEM high glucose supplemented with 10% FCS, 1× MEM Non-Essential amino acids, 20 μg/mL gentamicin (Thermo Fisher Scientific), referred to as complete medium at 37°C and 5% CO_2_. Both DZ and N91 are *MYCN*-amplified cell lines, and AS and SH are *MYCN*-nonamplified.

Blood samples were collected from adult healthy volunteers (French national blood service, EFS) who gave written informed consent in accordance with the Declaration of Helsinki [cell collection approved by the French Ministry of Higher Education, Research and Innovation (Codecoh: GRE-DC-2019-3803)]. Peripheral blood mononuclear cells (PBMC) were isolated by density gradient on lymphocyte separation medium, and cryopreserved until use, by standard freezing methods [37.5% FCS, 10% DMSO (Sigma-Aldrich)].

### Detection of the Chemokines Secreted by the Neuroblastoma Cell Lines

Supernatants from AS, SH, DZ, and N91 neuroblastoma cell lines were collected from 24-hour confluent cell cultures, and stored at −20°C before being used. Secreted chemokines were analyzed using human chemokine antibody array membranes (Abcam) which allow the detection of up to 38 chemokines, using a charge coupled device camera (exposition time of 90 seconds). A control with culture medium alone was performed to define secreted chemokines over the background of medium (Supplementary Fig. S1). The results were analyzed using the BIO-1D software. Comparison of samples was performed after normalization of spot intensity (AUC) using background and positive control signal intensities for each membrane and sample according to the formula:



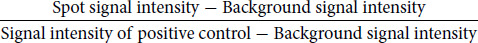



Cytometric beads arrays (BD Biosciences) were used to quantify CCL2, CXCL8, and CXCL10 (that were detected in the arrays) in the supernatants from 24-hour confluent neuroblastoma cell lines cultures of four biological replicates harvested in two different experiments.

### Chemokines Secretions by DCs in the Presence of Neuroblastoma Cell Lines

DCs were purified from PBMC obtained from 3 healthy volunteers, with Pan-DC Enrichment kit (Miltenyi Biotec) and cocultured for 24 hours with neuroblastoma cell lines, in 24-well plate (1:1 ratio, 0.5 × 10^6^ of each cell types) in DMEM 10% FCS (2 mL), with or without R848 (1 μg/mL). CCL19 and CCL22 productions were measured by ELISA (Sigma-Aldrich) in the harvested supernatants.

### Immunophenotyping and Calcium Measurement

The analysis of MHC class I protein surface expression was performed with the FITC-conjugated mouse anti–HLA-ABC or the isotopic control IgG2a antibodies (Beckman Coulter). Immunophenotyping to measure CCR2 expression on immune cell subsets among PBMC from healthy donors was performed by using the following antibodies: CD4 (FITC), CD56 (PE), CD8 (PerCP-Cy5.5), CD3 (APC-H7), TCRγδ (BV421), CD45 (V500), Lineage (FITC), CD123 (PE), HLA-DR (PerCP), and CD11c (BV421), purchased from BD Biosciences, and CCR2 (APC) from BioLegend. The panels used were designed to identify T cells (CD4^+^, CD8^+^, and γδ subsets), NK cells, MDC, and PDC. On the basis of the differential expression of CD11c among Lin+HLA-DR+ cells, we were also able to isolate monocytes and B cells (see Supplementary Fig. S2 for gating strategy). Percentages and median fluorescence intensity of CCR2 were analyzed. For the study of intracytoplasmic calcium concentration variations in immune cells, the following antibodies were used: Lin (FITC), HLA-DR (APC-H7), CD11c (BV421), and BDCA4 (APC, Miltenyi Biotec), to identify MDC and PDC, but also monocytes and B cells, as described previously, while NK and T cells were considered to be the majority of cells among Lin+ HLA-DR− cells (see Supplementary Fig. S3 for gating strategy). The method from Wendt and colleagues (23) was slightly adapted with slight modifications: PBMC (10^7^ cells/mL) were loaded with 1 μmol/L Fura-Red (Thermo Fisher Scientific) for 30 minutes in Hank's Balanced Salt Solution (HBSS with 1.25 mmol/L CaCl_2_, 0.5 mmol/L MgCl_2_; Gibco), washed and resuspended at 10^7^ cells/mL. After staining 15 minutes with antibodies, cells were washed, resuspended at 10^7^ cells/mL in HBSS before acquisition on the FACs. Fluorescence of Fura-Red was recorded at 510 and 760 nm upon excitation with the violet and blue laser, respectively, and the ratio 510/760 fluorescence over time was plotted with the FlowJo software. CCL2 (65 ng/mL; R&D Systems) was added after baseline recording for 25 seconds. Vehicle (PBS) and ionomycin (1 μg/mL; Sigma) were used as negative and positive controls, respectively.

Flow cytometry experiments and analyses were performed using an 8-color FACSCanto II flow cytometer with the Diva 8 or the FlowJo software (BD Biosciences).

### Migration Assays

The analysis of immune cells migration toward neuroblastoma cell lines was performed using transwells in 24-well plates equipped with inserts bearing 5 μm pores allowing cells active migration. A total of 5 × 10^5^ PBMC were seeded, and after a 2-hour incubation (37°C, 5% CO_2_), the migrating cells were collected, washed, resuspended in 150 μL, and 15 μL of fluorescent CountBright counting beads (Thermo Fisher Scientific) were added to perform cell numeration during flow cytometry acquisition. The cell suspension was then incubated with the following antibodies: CD4 (FITC), CD56 (PE), CD8 (PerCP-Cy5.5), CD3 (APC-H7), TCRγδ (BV421), CD45 (V500), Lineage (FITC), CD123 (PE), HLA-DR (PerCP), CD19 (APC), CD14 (APC-H7), and CD11c (BV421), purchased from BD Biosciences (see Supplementary Fig. S4 for gating strategy). The cells were then fixed with BD FACS Lysing Solution (BD Biosciences) and analyzed with a FACSCanto II using the BD FACS Diva software, and further analyzed with FlowJo or Diva, and treated using the GraphPad Prism software (GraphPad). The quantification of the absolute numbers of each immune cell subset for the migration assay was performed using this formula:



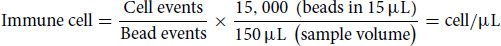



The proportion of each cell subset migrating compared with the total number of cell put in the insert was calculated by dividing the absolute numbers of migrating cells by the absolute number of the corresponding subset in the inserts. A positive control using recombinant human CCL2 (50 ng/mL, R&D Systems) was included, and blocking antibody directed against CCL2 (10 μg/mL, R&D Systems) was used to analyze the role of this chemokine in cell migration.

The analysis of T cells migration toward DCs supernatants was performed using the same protocol with slight modifications (18-hour incubation). Migrating T cells were quantified by flow cytometry with the following antibodies: CD4 (PE), CD8 (APC), and CD3 (BV421) purchased from BD Biosciences.

### Microarray and RNA Sequencing Analysis

Gene expression data from the Gene Expression Omnibus (GEO) dataset GSE62564 was downloaded from GEO (https://www.ncbi.nlm.nih.gov/geo/query/acc.cgi?acc=GSE62564) as log_2_RPM. The same set of samples is submitted under GEO accession GSE49711. The GSE62564 dataset is a reanalysis of the data. It contains transcriptional data from 498 neuroblastoma primary tumors. *MYCN* amplification status was extracted from the GSE49711 series matrix file. Because the *MYCN* amplification status of some tumors was unknown, we adopted the convention of naming *MYCN*-high or -low if log_2_RPM were above or below 8, as most *MYCN*-high neuroblastoma had *MYCN* amplification (Supplementary Fig. S5).

The GEO datasets GSE3960 and GSE85047 were downloaded from the GEO at NCBI (https://www.ncbi.nlm.nih.gov/geo/query/acc.cgi?acc=GSE3960). In GSE 3960, 101 primary neuroblastoma tumors were selected for quantification by microarray (24), while in GSE85047, 283 primary untreated neuroblastoma tumors were analyzed. *MYCN* amplification status was extracted from the series matrix files. For differential gene analysis or representation, raw data were Robust Multi-array Average normalized.

Spearman correlation analyses were performed in R. Because sample sizes and sequencing technologies were different between datasets, threshold for high correlation were defined individually for each dataset. For each gene g, a “high correlation” was defined as a correlation coefficient equal to or higher than 95% of correlations between the gene g and all other genes in the dataset.

We used strong correlations to build a mechanistic model. Strong correlations of immune cell markers with chemokine expression may be due to random effects or to unknown underlying confounding variable(s). Alternatively, they may reflect direct cell attraction by the chemokine or chemokine secretion by the cell, an interpretation that we favored when strengthened by existing biological knowledge. By selecting high correlations in two datasets that are biologically supported and for unidirectional interactions (i.e., cells secrete chemokine but do not respond to them, or chemokines attract cells but do not secrete them), we propose a stepwise model for T-cell recruitment.

For modeling and prediction of T-cell recruitment by chemokines, we applied penalized logistic regression (LASSO) with *CD3E* as binary response variable and a set of 34 chemokines as predictors (dataset GSE62564), to select the best set of chemokines for prediction. *CD3E* expression was classified as high or low (above or below median expression in each dataset). Gene expression data were z-transformed before training and testing the model. The tuning parameter lambda was selected by 10-fold cross-validation, and cross-validation was repeated 100 times. The mean coefficient for each chemokine was chosen for the final model and for testing datasets. The package glmnet in R (25) was used for training the model and package pROC (26) for ROC curve and AUC calculations.

A logistic regression model predicting *CD3E* and incorporating only the chemokines *CCL19* and *CCL22* was trained on the dataset GSE62564 using the glm function in base R and tested on datasets GSE3960 or GSE85047. The same analyses were repeated with *CD3D*, *CD3G,* and *CD247* (CD3 zeta) as T-cell markers, with similar results.

### Immunomodulation in the Presence of Neuroblastoma Cell Lines

We analyzed the maturation of DC upon TLR triggering in a two-dimensional coculture system, with cultures performed in the presence or not of neuroblastoma cell lines (50% confluent cells). Frozen PBMC from healthy donors were thawed, and cultured at 10^6^ cells/mL, in 24-well culture plates, for 24 hours with or without TLR ligands [R848 (1 μg/mL), CpGA ODN-2336 (1.5 μmol/L) or LPS (from *E. coli* K12, 0.1 μg/mL) from Invivogen]. Supernatants were then harvested and frozen. IL12p70, IFNα2, TNFα, IL6, and IL8 concentrations were measured by Cytometric Bead Array (CBA) Technology (BD Biosciences).

### Statistical Analysis

Statistical analyses were performed by GraphPad Prism software (GraphPad). Statistical significance was determined by indicated adequate tests (Student *t* test or Mann–Whitney to compare two groups, one-way ANOVA nonparametric Friedman test with Dunn test for more than two groups comparison, paired two-way ANOVA with Bonferroni test for more than two groups comparison. Linear mixed modeling was performed to assess the significance of *MYCN* amplification for CCL2 secretion and DC migration, with *MYCN* status as fixed effect and random intercepts for cell lines. *P* values were obtained by likelihood ratio tests. These analyses were performed using R and the lme4 package (27). Figures were generated in GraphPad Prism or in R with the tidyverse package (28) and the pheatmap and ComplexHeatmap (29) packages.

### Data Availability

The data generated in this study are available within the article and the Supplementary Data. Data analyzed were obtained from GEO at: https://www.ncbi.nlm.nih.gov/geo/query/acc.cgi?acc=GSE62564, https://www.ncbi.nlm.nih.gov/geo/query/acc.cgi?acc=GSE3960, and https://www.ncbi.nlm.nih.gov/geo/query/acc.cgi?acc=GSE85047.

## Results

### *MYCN*-Amplified Neuroblastoma has a Reduced Ability to Recruit Monocytes and DCs

**FIGURE 1:**
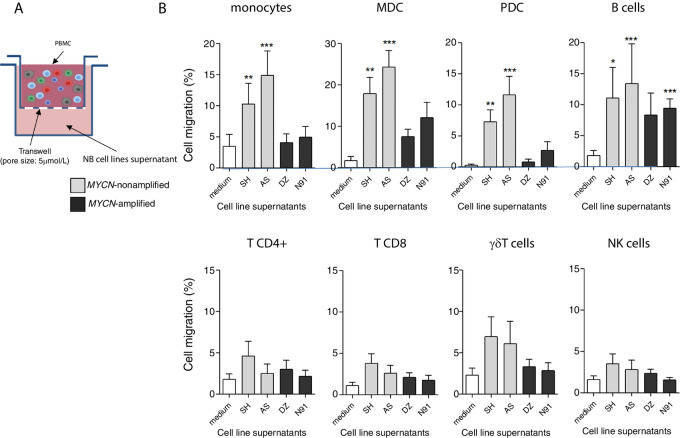
*MYCN*-amplified neuroblastoma has reduced ability to recruit monocytes and DCs. **A,** Experiment presentation: PBMC were seeded in transwell inserts with 5-μm pores, in wells containing supernatants from neuroblastoma cell lines in the bottom chamber. After 2 hours, migrating cells were harvested, labeled to identify immune cell subsets, and counted by flow cytometry using Count Bright beads. **B,** Percent migration was calculated, relative to absolute number of input cells. Results are presented for each cell type, mean ± SD of three experiments, each performed with 3 different healthy donors cells (*N* = 9 healthy donors). Friedman nonparametric statistical analysis was used, with Dunn test, to identify significant differences between groups. *, *P* ≤ 0.05; **, *P* ≤ 0.01; ***, *P* ≤ 0.001.

*In vitro* migration assays of PBMC toward supernatants of *MYCN*-amplified (DZ and N91) and -nonamplified (AS and SH) neuroblastoma cell lines were performed (Fig. 1A). Absolute numbers of migrating cells were counted, and the percentage of migrating cells was calculated for each cell subset. As shown in Fig. 1B (top), the supernatants from *MYCN*-nonamplified neuroblastoma were highly chemoattractive for monocytes, MDC and PDC, with more than 10%, 18%, and 7% of migrating cells respectively (in 2 hours), compared with the supernatants from *MYCN*-amplified neuroblastoma that did not induce any significant migration. B cells significantly migrated toward three of four neuroblastoma cell lines supernatants, independently of *MYCN* amplification status. Effector cell (T, NK, γδ T cells) migration was lower, and not significantly different from the migration observed in the control condition (Fig. 1B, bottom). Therefore, neuroblastoma with *MYCN* amplification display an impaired ability to recruit monocytes, MDC and PDC, in contrast to *MYCN*-nonamplified neuroblastoma.

### *MYCN*-Nonamplified Neuroblastoma Displays High Secretion of CCL2, While its Receptor CCR2 is Expressed by Monocytes, Myeloid, and PDCs

**FIGURE 2:**
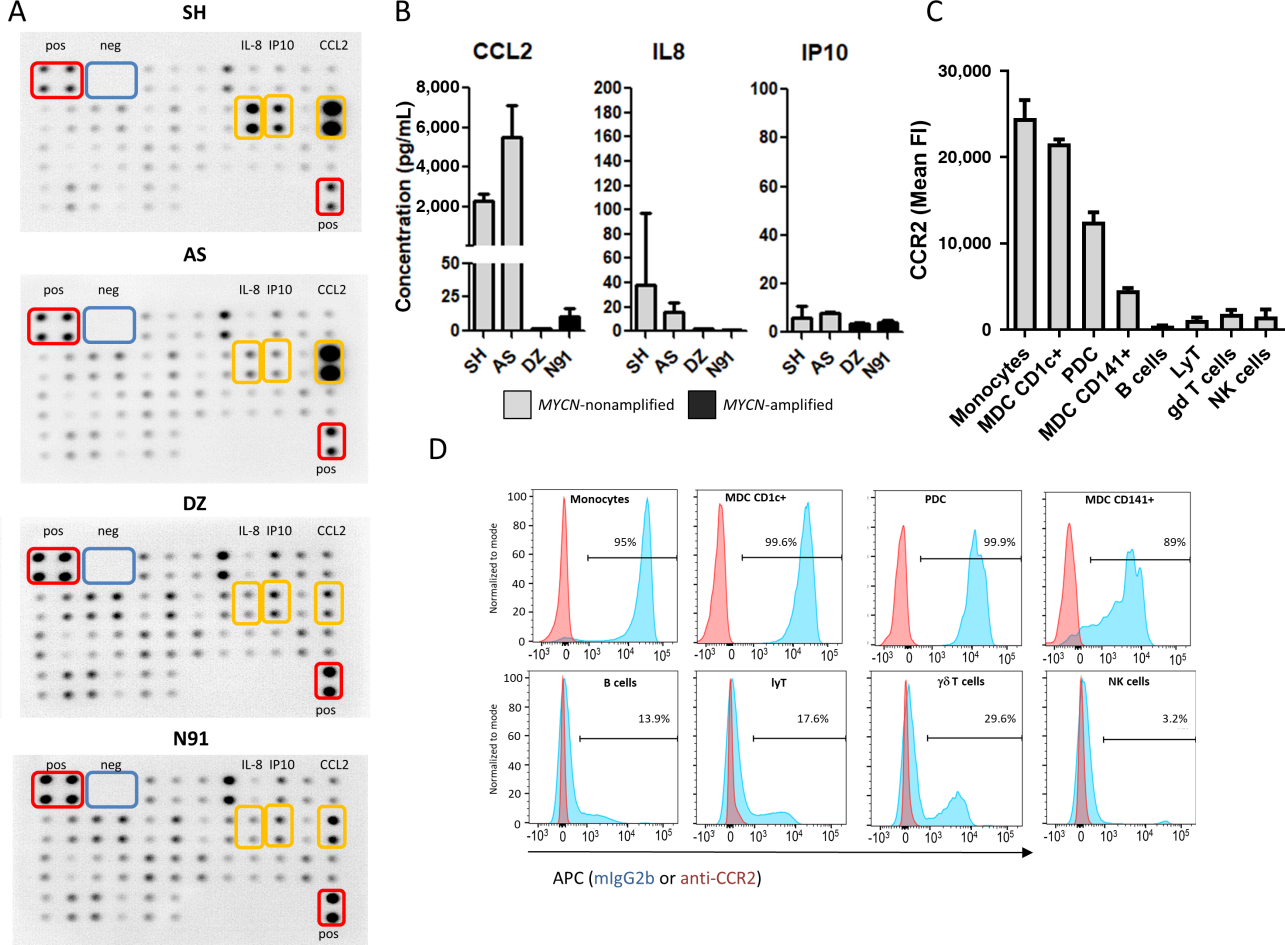
Neuroblastoma cell lines secrete few chemokines but CCL2, whose ligand CCR2 is expressed by monocytes, myeloid and plasmacytoid DCs. Chemokine arrays were used to detect chemokine secretions in supernatants recovered from confluent neuroblastoma cell lines that were incubated 24 hours. **A,** Raw image showing the intensities of the spots corresponding to the 38 targeted chemokines. A single experiment was performed. **B,** CXCL8, CXCL10, and CCL2 were measured by cytometric beads arrays in cell culture supernatant from the four neuroblastoma cell lines. Bars show the mean ± SD of four independent cultures. **C,** Mean fluorescence intensity of CCR2 expression was measured on PBMC from 2 healthy donors, and bars show the median and range of these two measures. **D,** Representative experiment (out of two) showing CCR2 expression analyzed by flow cytometry on PBMC, to define which cell subsets expressed this receptor. Blue histograms represent background with control antibody, and red histogram the specific labeling with CCR2 antibody.

To identify the chemokines responsible for the strong attraction of monocytes and DC by *MYCN*-nonamplified neuroblastoma, we looked for the presence of 38 chemokines (Supplementary Fig. S1). Raw data of the arrays for neuroblastoma supernatants are shown in Fig. 2A, and Supplementary Fig. S1A displays the data for culture medium. After normalization, only three chemokines were detected at higher level in one or two supernatants, compared with culture medium, namely IL8/CXCL8, IP-10/CXCL10, and MCP-1/CCL2. These cytokines were then quantified in supernatants from four biological replicates of these cell lines, by cytometric beads arrays (Fig. 2B). Low level of CXCL8 was secreted by the SH cell line, while CXCL10 level was below 10 pg/mL for all cell lines. Interestingly, CCL2 was secreted at high level by the *MYCN*-nonamplified AS (mean = 2,329 pg/mL) and SH (mean = 5,503 pg/mL) neuroblastoma cell lines, but was not found in the supernatants of *MYCN*-amplified neuroblastoma cell lines (N91 and DZ), suggesting that this chemokine could be involved in the differential recruitment of monocytes and DC we observed. A linear mixed model analysis applied to these data confirm the statistical difference of CCL2 secretion between *MYCN*-amplified or not cell lines (*P* < 0.05, likelihood ratio test).

The expression of CCR2 (CCL2 receptor) was analyzed on immune cell subsets that were gated as depicted in supplementary Fig. S2. CCR2 was expressed by more than 90% of monocytes, MDC and PDC with a median fluorescence intensity above 1,000 or 10,000 (Fig. 2C and D). Conversely, CCR2 was expressed at very low levels by B cells, T lymphocytes (CD4^+^, CD8^+^, γδ^+^), or NK cells. These data suggest that CCL2 may be responsible for the recruitment of CCR2-expressing monocytes, MDC and PDC by *MYCN*-nonamplified neuroblastoma.

### Monocytes, MDC, and PDC Migration Toward *MYCN*-Nonamplified neuroblastoma Cell Lines Is Driven by the CCL2/CCR2 Axis

**FIGURE 3:**
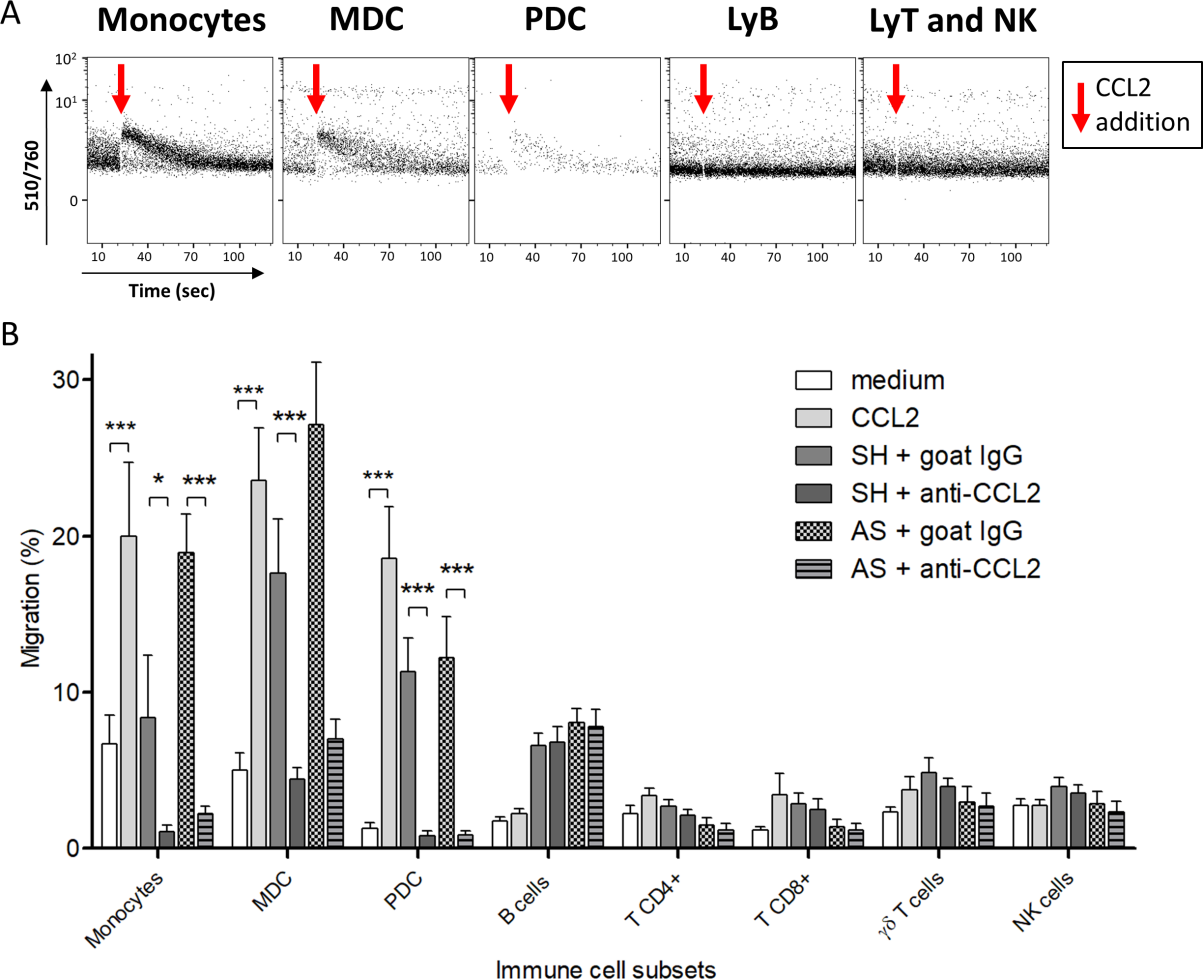
CCL2 induces a calcium influx in DCs, and is responsible for DC recruitment toward neuroblastoma. **A,** Signaling induced by CCL2 in cell subpopulations in PBMC was measured by calcium mobilization using the Fura-Red probe. Cells were loaded with Fura-Red and stained to gate specific immune cells. Calcium mobilization upon CCL2 addition (65 ng/mL) was measured by an increased fluorescence ratio (510 nm/760 nm). CCL2 induces calcium mobilization in monocytes, MDC and PDC, but not in B lymphocytes, T cells, or NK cells. Data from one representative experiment out of three. **B,** Transwell migration (in 2 hours) of immune cells from PBMCs toward medium, medium with CCL2, or culture supernatant from the cell lines SH and AS. CCL2 neutralizing antibody or isotype control was added to measure CCL2-driven migration. Values represent the percentage of initial cells migrating in 2 hours. Bars represent mean ± SD of two experiments, performed with 3 and 5 different healthy donors’ cells (*N* = 8 healthy donors).

The role of CCL2/CCR2 axis in immune cell recruitment was studied further, and we evaluated intracytoplasmic calcium mobilization by analyzing Fura-Red fluorescence in CCL2-exposed cells, to verify the functionality of the receptor for each cell type. We observed that addition of CCL2 to PBMC was immediately followed by an increase of intracytoplasmic calcium concentration in monocytes, MDC, and PDC, but not in other cell subsets (Fig. 3A; Supplementary Fig. S3), in accordance with differential CCR2 expression (Fig. 2C and D). To further demonstrate the functionality of CCR2, migration experiments were performed. As shown in Fig. 3B, among PBMC, CCL2 was a highly efficient chemoattractant for monocytes, MDC and PDC, with more than 20% of migrating cells counted after 2 hours. Conversely, the migration of B cells, T cells, or NK cells was very low, and of the same magnitude as toward medium alone, in agreement with their low CCR2 expression.

Importantly, when supernatants from the *MYCN*-nonamplified neuroblastoma cell lines SH and AS were tested for their ability to attract immune cell subsets, we observed that migration of monocytes, MDC and PDC induced by these supernatants was abrogated by the addition of anti-CCL2 antibodies, while the modest migration of the other cell subsets was not affected (Fig. 3B). By using linear mixed model analysis taking into account the source of PBMC, the cell line and their *MYCN*-amplification status, we confirmed that the migration of monocytes, MDC, and PDC cells was statistically higher toward *MYCN*-nonamplified neuroblastoma cell lines supernatants (*P* < 0.05, likelihood ratio test). Altogether, these results demonstrate the major involvement of the CCR2/CCL2 axis in the recruitment of APC by *MYCN*-nonamplified neuroblastoma.

### CCL2-Driven Recruitment of CCL19- and CCL22-Producing CD1c^+^ DCs may Induce T-Cell Recruitment in Neuroblastoma Tumors

**FIGURE 4:**
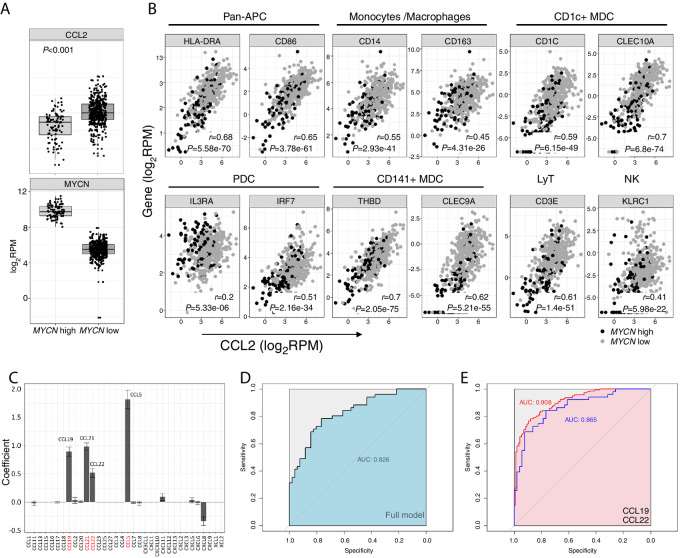

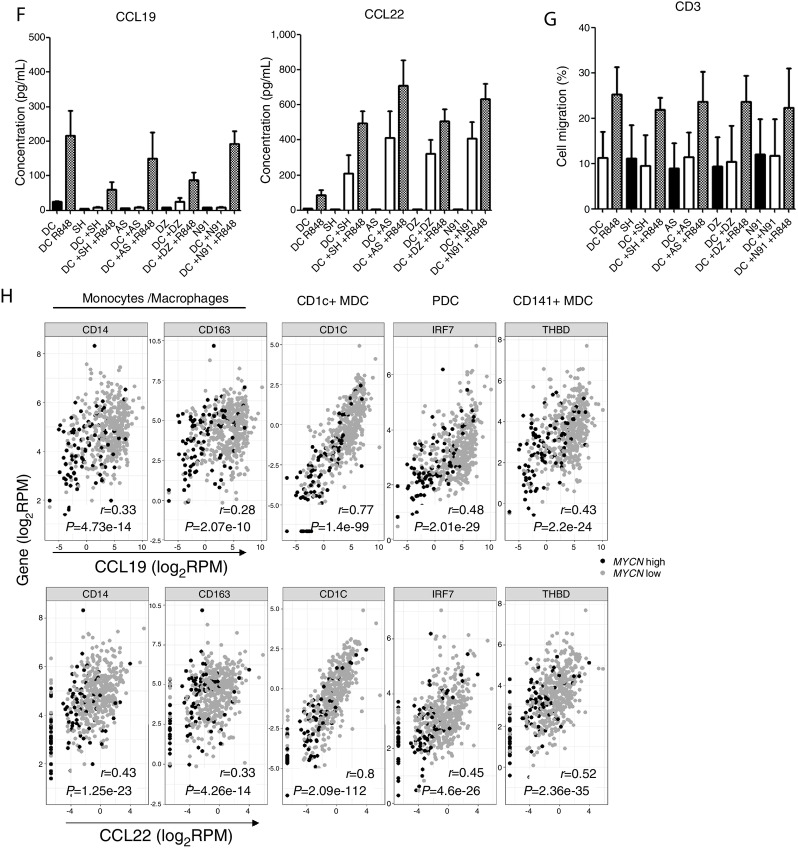
*CCL2* level in neuroblastoma tumors correlates with DC infiltrate, role of CCL19, CCL22, and CD1c^+^ DC in T-cell recruitment. **A,** Normalized expression of *CCL2* and *MYCN* genes in the GSE62564 dataset. Tumors are separated according to the genetic amplification of *MYCN* (i.e., low or high). Statistical analysis: Student *t* test. **B,** Correlation plots between *CCL2* expression and genes coding for specific immune population markers (specified above plots). High correlations (see Materials and Methods) in the dataset are marked in bold (Spearman correlation coefficient *r* and *P* value). **C,** Penalized logistic regression coefficients of 34 chemokines for regression of *CD3E* in the dataset GSE62564 (mean ± SD of 100 × 10-fold cross-validations). **D,** ROC curve and AUC of the full model defined in **C**, tested on dataset GSE3960. **E,** ROC and AUC of a logistic regression model of *CD3E* versus *CCL19* and *CCL22* trained on GSE62564 and tested on GSE3960 (blue) or GSE85047 (red). **F,** CCL19 and CCL22 were measured by ELISA in supernatants from cocultures of the four neuroblastoma cell lines and purified DCs activated or not by R848. Bars show the mean ± SD of three independent cultures. **G,** Transwell migration (18 hours) of immune cells from PBMCs toward medium, or supernatant from cocultures of the four neuroblastoma cell lines with or without DCs activated or not with R848. Values represent the percentage of initial CD3^+^ T cells migrating in 18 hours. Bars represent mean ± SD of three experiments, performed with different healthy donors’ DCs. **H,** Correlation plots between *CCL19* and *CCL22* expression and genes coding for specific immune population markers. High correlation in the dataset is marked in bold (Spearman correlation coefficient *r* and *P* value). *MYCN* status is indicated for each tumor (gray: *MYCN*-low, black: *MYCN*-high).

To further confirm the role of CCL2/CCR2 axis in APC recruitment to neuroblastoma tumors, we used the public RNA sequencing (RNA-seq) dataset GSE62564 (30) containing data from 498 samples among which 91 had a high expression of *MYCN*. The GSE3960 and GSE85047 microarray datasets, containing 102 samples among which 20 were *MYCN* amplified (GSE3960; ref. 24), or 283 primary neuroblastoma of which 55 were *MYCN* amplified (GSE85047), were used to test our findings. We first observed a lower level of expression of *CCL2* in *MYCN*-high neuroblastoma tumors (Fig. 4A, *P* < 0.001; Supplementary Fig. S6A), likely reflecting the repression of *CCL2* expression by the *MYCN* oncogene, but some *MYNC* low neuroblastoma had low levels of *CCL2* transcription, and the reverse was also true. Although causal relationships are difficult to prove in observational studies, we propose here a working model for T-cell recruitment in neuroblastoma tumors, based on our experimental data, the known biology of chemokines, gene correlation analysis, and predictive models (see Materials and Methods for discussion).

The correlation between *CCL2* gene expression level and specific immune cell subsets markers was evaluated in GSE85047. Besides *CD14* and *CD163* (monocytes/macrophages), *CD3E* (T cells), *KLRC1* (NK cells) and *HLA-DR* and *CD86* (Pan-APC), a short list of genes was defined, allowing identification of DC subsets, based on Villani and colleagues published data (31). The chosen genes were *CD1C* and *CLEC10A* for CD1c^+^ MDC subset, *IL3RA* and *IRF7* for PDC, and *THBD* and *CLEC9A* for BDCA3/CD141^+^ MDC subset. As shown in Fig. 4B, there was a high correlation (*r* ≥ 0.54 and *P* < 0.001, above 5% threshold, see Materials and Methods) between *CCL2* expression and pan-APC, CD1c^+^ MDC, and CD141^+^ MDC markers. A lower correlation was observed between *CCL2* level and monocytes/macrophages, and PDC (*IRF7*) infiltration, suggesting the involvement of CCL2/CCR2 in APC recruitment by neuroblastoma tumors. These results were confirmed in the GSE3960 microarray dataset (Supplementary Fig. S6). Intriguingly, a *CCL2/ CD3E* correlation was also observed (*r* = 0.61, *P* < 0.0001). As CCR2 is poorly expressed by circulating T cells, and CCL2 does not attract T cells in our migration experiments, we looked for an indirect link. We analyzed univariate correlations between T-cell infiltration (*CD3E* level) and the level of expression of 34 chemokines, in the two datasets. As shown in the Supplementary Fig. S7A correlation maps, *CCL5*, *CCL19, CCL21,* and *CCL22* levels were highly correlated with *CD3E* levels, more so than any other chemokine. These high univariate correlations are also shown in Supplementary Fig. S7B, with *r* > 0.8 in GSE62564, and *r* > 0.4 in GSE3960 (*P* < 0.001). In another approach, we used penalized logistic regression to build a model predicting *CD3E* levels (high vs. low) with a minimal set derived from 34 chemokines. By shrinking some coefficient estimates toward zero, the procedure essentially selects a subset of chemokines for regression, leading to a model with potentially good predictive accuracy. In this instance, the LASSO trained on GSE62564, retained *CCL19, CCL21, CCL22, CCL5,* and *CXCL8* as the main variables for predicting *CD3E* levels (Fig. 4C). This full logistic model performed well when tested on GSE3960, despite different technologies used to acquire data in the training and testing datasets (Fig. 4D). Among the selected chemokines, CCL5 can be produced by T cells, and CCL21 is mainly produced by stromal cells, while CCL19 and CCL22 can be produced by DCs or monocytes/macrophages (32–36). A conventional logistic regression model trained on GSE62564 and including only these two chemokines exhibited a very good discriminating power when tested on GSE3960 and GSE85047 (Fig. 4E).

To confirm the involvement of CCL19 and CCL22 in T-cell migration, we analyzed CCL19 and CCL22 secretion by DCs (comprising PDC, cDC1, and cDC2), purified from healthy donors blood, that were cocultured for 24 hours in the presence or not of neuroblastoma cell lines and activated or not. We observed CCL19 production after R848 activation of DC in the presence or not of neuroblastoma cell lines. On another hand, CCL22 was secreted by DC that were in contact with neuroblastoma cell lines, even in the absence of R848, and there was an additive effect of neuroblastoma cell lines and R848 on CCL22 production by DC (Fig. 4F). Migration experiments were then performed, to analyze T-cell recruitment by these supernatants. Enhanced T-cell migration was driven by supernatants obtained from activated DC, and was not modified by the presence of neuroblastoma cells (Fig. 4G). Basal migration of CD4^+^ T cells (20%) was higher than basal migration of CD8^+^ T cells (2%), but both subsets were attracted by activated DC culture supernatants (Supplementary Fig. S7C). Altogether, these data suggest that CCL19 and CCL22 produced by activated DC play an important role in recruiting T cells to the neuroblastoma microenvironment *in vitro* and *in vivo*.

To decipher which APC may be responsible for CCL19 and CCL22 secretions in neuroblastoma tumors, we analyzed correlations between these chemokine levels and APC-specific markers. *CD1C* was highly correlated with *CD3E* in both datasets (*r* > 0.5 in GSE3960, and *r* > 0.8 in GSE62564, *P* < 0.001; Supplementary Fig. S7D). Both *CCL22* and *CCL19* expression levels were highly correlated with the level of *CD1C* in both dataset (*r* > 0.7 in GSE62564 and, *r* > 0.4, in GSE3960 *P* < 0.001; Fig. 4H; Supplementary Fig. S7E), and *CCL22* was correlated with *CD141*. Altogether, our results suggest that CCL2 produced by neuroblastoma cells initiate the recruitment of monocytes, myeloid and plasmacytoid DCs, and that among these cells, the CD1c^+^ subset when activated may recruit T cells by means of CCL19/CCL22 secretion.

Besides recruitment of immune cells, the efficacy of antitumor immune responses also relies on the correct processing and presentation of antigens by the tumor cells. We measured the expression of HLA class I molecules at the surface of the neuroblastoma cell lines. As described already (37–39), we observed that *MYCN*-amplified neuroblastoma cells lines (DZ and N91) lacked expression of HLA class I molecules, whereas AS and SH expressed it at high level (Supplementary Fig. S8A). On a heatmap, we represented the expression of *CCL2, CCL19, CCL22*, and a set of genes coding for proteins expressed by APC subsets, by T cells, and for molecules associated with HLA class I presentation pathway for GSE62564 (Supplementary Fig. S8B). Samples were separated according to *MYCN* amplification status and sorted on the basis of *CCL2* level. *CCL2* and HLA class I genes were found more often downregulated in neuroblastoma tumors with *MYCN* amplification. In agreement with our *in vitro* data, we observed an increased representation of DC-associated genes in neuroblastoma with higher *CCL2* expression, further illustrating the role of CCL2 in the active recruitment of DCs.

### Plasmacytoid DC Activation Is Enhanced in *MYCN*-Nonamplified Neuroblastoma Cell Lines Microenvironment

**FIGURE 5:**
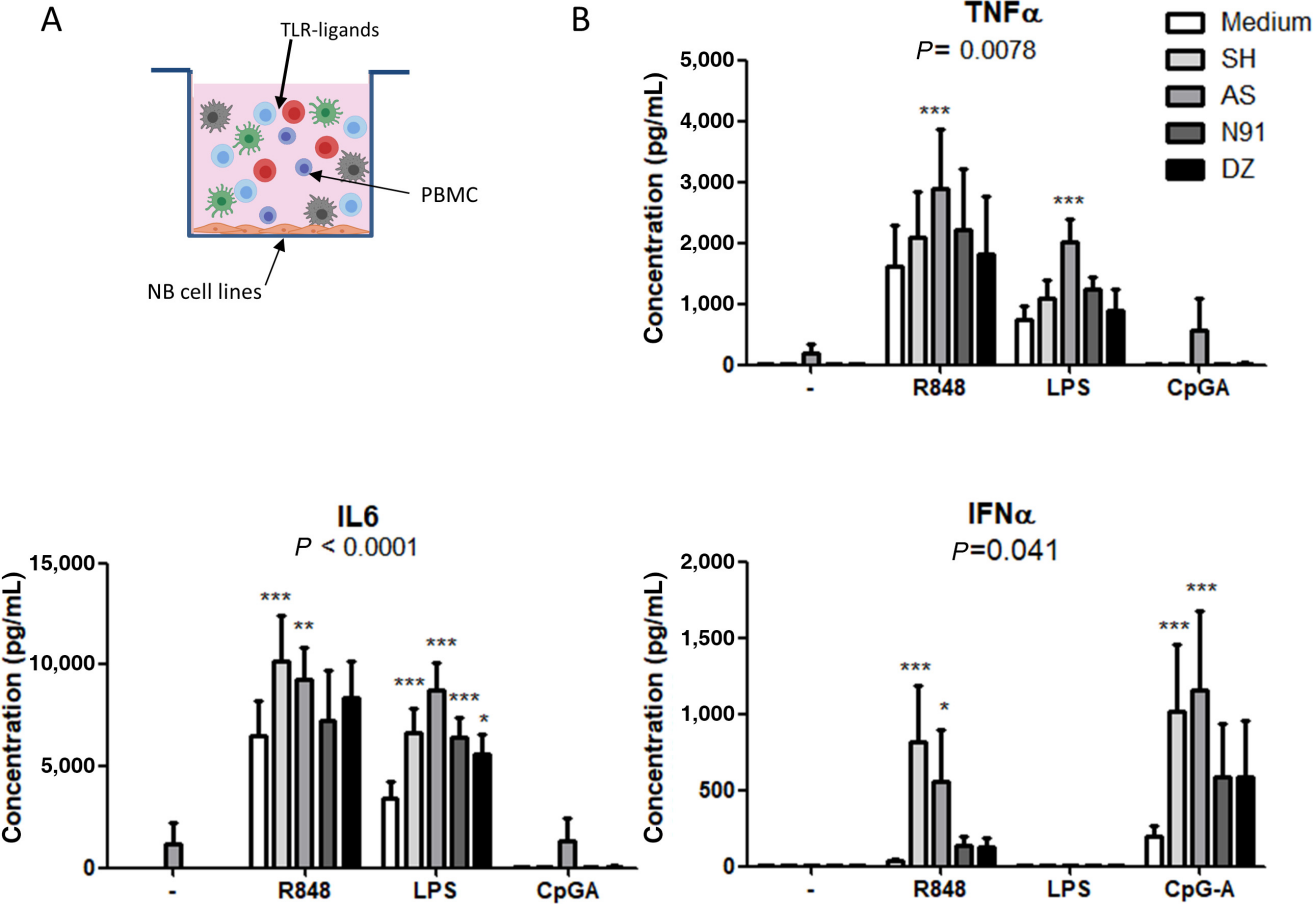
Modulation of DC functionality in the presence of neuroblastoma cell lines. Immune cell functionality was evaluated by activating PBMC from HD (*n* = 7) with R848 or LPS in the presence or not of neuroblastoma cell lines. **A,** Experiment presentation: PBMC were seeded in wells containing 50% confluent neuroblastoma cell lines. After 24 hours, supernatants were harvested, and cytokine content was measured by Cytometric Bead Array. **B,** Cytokine secretions measured in harvested supernatants. Statistical analysis: paired two-way ANOVA with Bonferroni post-test to compare with medium culture.

To analyze the potential immune modulation induced by neuroblastoma, cocultures of neuroblastoma cell lines with immune cells were performed, and the capacity of DCs to respond to activation by TLR ligands in this environment was evaluated (Fig. 5A). R848 (TLR7/8 ligand), LPS (TLR4 ligand), and CpG-A (TLR9 ligand) were chosen to evaluate the activation of both MDC and PDC. TNFα was secreted after activation with LPS and R848, and the highest secretion of this cytokine was observed when immune cells were cocultured with the AS cell line. Concentrations of IL12p70 were very low (≤20 pg/mL) in all activated conditions, while concentrations of IL8 were very high (≥10 ng/mL) after activation with R848 or LPS, but no difference was observed between cultures performed in presence or absence of neuroblastoma cells (Supplementary Fig. S9). As shown in Fig. 5B, upon activation with R848, a significantly increased secretion of IL6 was observed in the cocultures performed with the *MYCN*-nonamplified cell lines AS and SH, while IL6 secretion was enhanced after LPS activation in cocultures with the four neuroblastoma cell lines. Strikingly, secretion of IFNα, which is a cytokine specifically produced by PDC upon R848 or CpG-A activation, was significantly increased when immune cells were in contact with the *MYCN*-nonamplified neuroblastoma cell lines AS and SH, suggesting that these cell lines, but not the *MYCN*-amplified cell lines, were able to create a microenvironment favoring plasmacytoid DC activation.

## Discussion

Neuroblastoma is a frequent tumor in childhood arising from the sympathetic nervous system. The most aggressive subset of neuroblastoma has been associated with recurrent somatic mutations and with *MYCN* oncogene amplification, which can modulate antigens expressed in tumor cells and thus influence immune surveillance (3). Primary metastatic neuroblastoma with *MYCN* amplification is associated T-cell-poor microenvironment (15).

In the current study, the recruitment and the migration of immune cells induced by neuroblastoma cell lines with *MYCN* amplification or not were analyzed. As opposed to *MYCN*-amplified cell lines, *MYCN*-nonamplified neuroblastoma was highly chemoattractive for monocytes, MDC and PDC. Effector cell (T, NK, and γδT cells) migration was lower compared with the migration of APC in this time frame (2 hours), and not significantly different from migration observed in the control condition. The presence of activated T cells within primary neuroblastoma and the possibility for autologous T cells to become activated in the presence of neuroblastoma cells has been demonstrated (40). Interestingly, there is a high correlation between T cells, NK cells, and DC infiltration in neuroblastoma tumors, suggesting a coordinated recruitment of these cells, which is associated with a favorable prognosis, independently on *MYCN*-amplification status (8). Conversely, reduced T-cell infiltration has been observed in primary neuroblastoma tumors with *MYCN* amplification, together with lower IFN pathway activity and chemokine expression (15), suggesting that *MYCN* amplification could impact immune cell recruitment.

In our study, we observed little chemokine secretion by neuroblastoma cell lines, except for CCL2, that was secreted by *MYCN*-nonamplified neuroblastoma cell lines but not by *MYCN*-amplified neuroblastoma cell lines, suggesting that this chemokine could be involved in the observed differential recruitment of monocytes, MDC and PDC. Indeed, CCL2 is involved in the regulation of DC precursor transit into peripheral tissues and for normal inflammatory monocyte migration into peripheral tissues (41).

We observed that monocytes, MDC and PDC express CCR2 at much higher levels compared with other immune cells, respond to CCL2 by a rapid calcium mobilization and are attracted by CCL2 in migration experiments, contrary to the other cell subsets that do not express CCR2. Blocking experiments demonstrate that CCL2 is responsible for these CCR2-expressing cells recruitment by *MYCN*-nonamplified neuroblastoma, as already observed for the migration of CCR2-expressing NKT cells toward CCL2-expressing neuroblastoma (20, 21).

DCs are pivotal cells for antitumor immune response priming (42). Their presence positively correlates with the clinical outcome of patients with neuroblastoma and is an independent predictor from those currently used to stage and stratify treatment of patients with neuroblastoma (8). Our data suggest that monocytes and DC recruitment is directly associated with CCL2 secretion by neuroblastoma cells, as already shown for iNKT (20, 21). Further investigation of the bidirectional cross-talks that may occur between APC and iNKT in neuroblastoma TME, could pave the way for development of new therapeutic strategies harnessing the biology of these cells (43). It has been shown that CCL2 expression is repressed in *MYCN*-amplified neuroblastoma cells (21), and our analysis using two different public transcriptomic datasets confirm that *CCL2* transcription was lower in *MYCN*-amplified neuroblastoma (which may arise from tumor or immune cells). We also observed a continuum of *CCL2* expression with low *CCL2* expression in some *MYCN*-nonamplified neuroblastoma, suggesting that other mechanisms could repress *CCL2* transcription. Epigenetic mechanisms could be involved in *CCL2* repression in neuroblastoma, as recently described in lung cancer, where epigenetic silencing of *CCL2* by DNMT1 and the EZH2/H3K27me3 axis potentiates tumor development by inhibiting macrophage infiltration (44). Neuroblastoma with low CCL2 expression may be unable to induce DC and iNKT recruitment, leading to immune escape by ignorance.

We observed that *MYCN*-amplified tumors expressed low levels of HLA-class I molecules, such defect could be linked to suppression of MHC-class I gene expression through downregulation of the p50 subunit of NFκB (45). This observation was also made at transcriptomic level in neuroblastoma tumors, with downregulation of mRNA encoding for HLA class I and molecules associated with Ag presentation, in *MYCN*-amplified tumors. However, we cannot decipher whether it results of MYCN direct effect on HLA-gene expression in tumor cells, or from an indirect effect due to CCL2 downregulation, resulting in low immune cell infiltration and inflammation. Immunosurveillance by Cytotoxic T Lymphocytes relies on correct MHC class I expression at the surface of tumor cells, allowing the specific recognition of malignant cells (17); however, tumor cells lacking MHC class I molecules are more sensitive to NK cell–mediated cytotoxicity. The composition of immune infiltrate in neuroblastoma is heterogeneous, some tumors being highly infiltrated by T lymphocytes and innate effector cells (iNKT, NKT, and γδ T cells), while other tumors are characteristic immunologically “cold” tumors. An inverse correlation between *MYCN* amplification and leukocyte infiltration has been demonstrated by *in silico* analysis (46), and the role of *MYCN* in immune network dysregulation has been described, leading to lower Th1 immunity (47). Our results suggest that CCL2 is crucial for the initial recruitment of monocytes, MDC and PDC by neuroblastoma, and paves the way for the further recruitment of effector cells in these tumors. Using transcriptomic public datasets, we found that 4 chemokines (CCL5, CCL19, CCL21, and CCL22) were strongly correlated with T lymphocyte infiltration. Both CCL19 and CCL22 can be produced by MDC after activation (36), there are however no data available for chemokines production by CD141^+^ MDC, certainly in relation with their very low numbers that make their study difficult. In the simplified model we propose, CCL19 and CCL22 are sufficient to predict T lymphocyte recruitment. Interestingly, CCL22 was found in supernatants of cocultured DC and neuroblastoma cells, but activation of DC was needed to induce CCL19 and T-cell migration in the same settings. In the TME of *MYNC*-nonamplified neuroblastoma, DC activation may be induced by tumor-derived damaged-associated molecular patterns (48), or through DC-iNKT cross-talk (49). CCL19 and CCL22 are ligands for T cells that express CCR7 (naïve and central memory stages of differentiation) or CCR4 (Th2 cells and regulatory T cell), so we propose that MDC, and more specifically the CD1c^+^ and CD141^+^ subsets can induce T-cell recruitment in neuroblastoma by means of CCL19 and CCL22. Interestingly, besides *MYCN* amplification that is a bad prognosis factor in neuroblastoma, a recent study found that transcription of five genes including *CCL19* and *CD1C*, could predict better prognosis (50).

**FIGURE 6:**
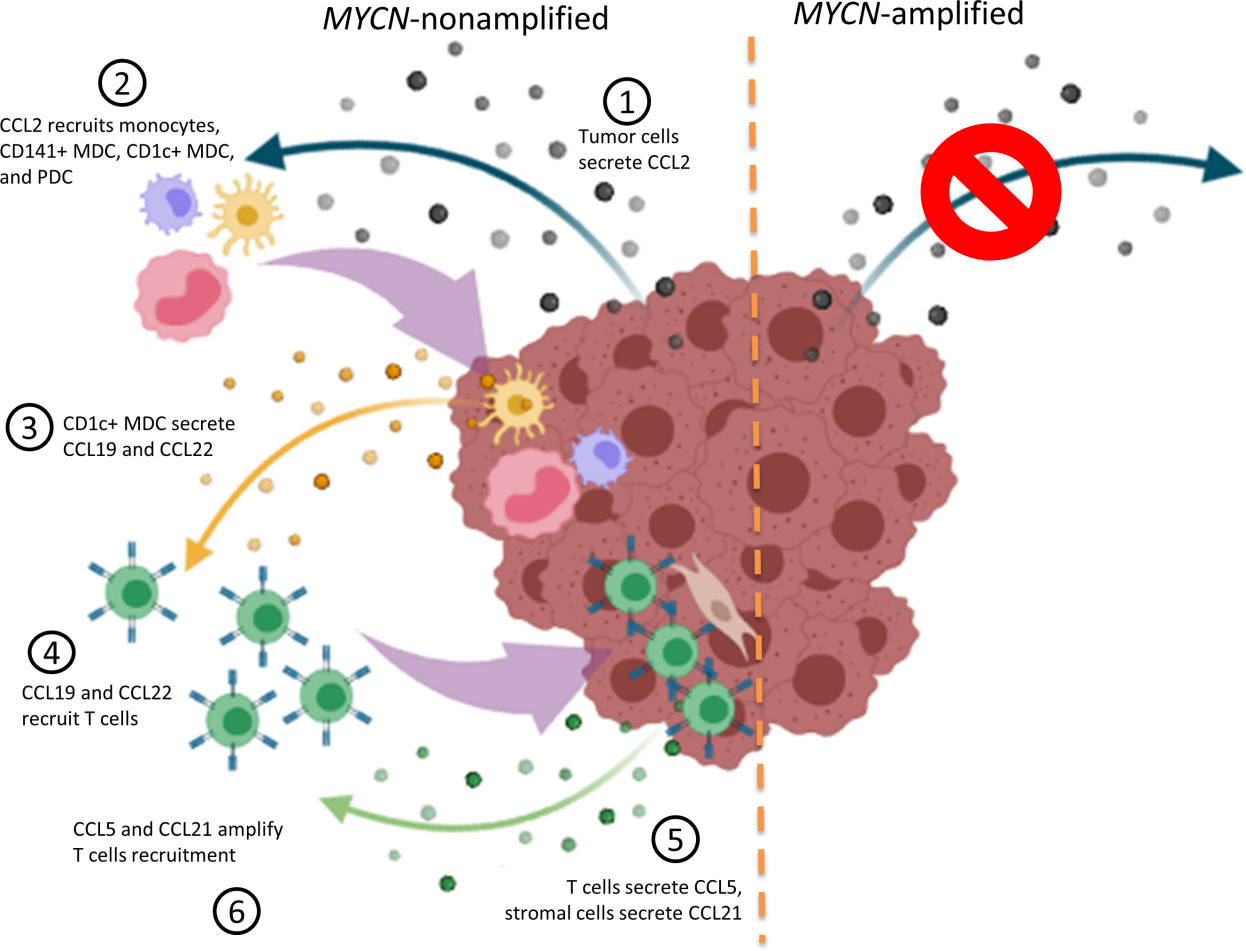
Proposed model of immune cell recruitment in neuroblastoma**.***MYCN*-nonamplified neuroblastoma (left side) secrete CCL2, a chemokine that will initiate recruitment of APCs: monocytes and DCs (CD141^+^ and CD1c^+^ MDC, and PDC). The TME in these tumors favors DC activation, and among them, CD1c^+^ MDC will secrete CCL19 and CCL22, thereby allowing T-cell recruitment. Immune infiltration is further amplified with CCL5 secretion by T cells, and CCL21 produced by stromal cells. In *MYCN*-amplified neuroblastoma (right side), in the absence of CCL2 production, the tumor remains “cold.” Created with BioRender.com.

We also looked at immunomodulation induced by neuroblastoma cell lines, and found that tumors without *MYCN* amplification create a microenvironment that favors the activation of DC. Indeed, when immune cells were cocultured with such neuroblastoma cell lines, an increased cytokine secretion was measured following DC activation. The large increase in IFNα secretion observed after activation with R848 and CpGA suggests that PDC reactivity is enhanced in *MYCN*-nonamplified TME. Such enhancement of immune response was not observed when immune cells were in contact with *MYCN*-amplified neuroblastoma cell lines. Because PDC can participate to activation of adaptive and innate immunity, any alteration of their function in the TME may greatly affect immune response. In the context of neuroblastoma, targeting PDC function to exploit their ability to activate NK cells may represent innovative approach in combined immunotherapy (51). To confirm the relevance of these results, it could be very interesting to analyze primary neuroblastoma—infiltrating DC function *ex vivo*, as we have already done in melanoma (52), as DC functions and metabolism are usually impaired in TME (53, 54). Here, we observed an inverse phenomenon, with a higher functionality of DCs in contact with *MYCN*-nonamplified neuroblastoma. Identifying factors involved in such adjuvant effect could lead to the development of new therapeutic strategies, by providing new ways to improve DC function in tumors. A recent review summarizes dual protumoral or antitumoral role of CCL2, that may depend on the interaction between cancer and immune cells (55). In neuroblastoma, CCL2 could be a candidate molecule that could favor DC function; indeed, emerging evidence points to a role for CCL2 in modulating immune cell function (cytokine secretion, adhesion, polarization; ref. 56). Decreased CCL2 expression in *MYCN*-amplified cell lines may also favor ineffectiveness of immune checkpoint blockade therapy, and being able to modulate immune cell infiltration in neuroblastoma tumors could result in transformation of “cold” to “hot” tumors, rendering them more sensitive to immunotherapies (Fig. 6). Epigenetic modifiers could be attractive drugs to restore a microenvironment more permissive for immune responses in *MYCN*-amplified neuroblastoma (57).

Overall, our findings highlighted a major role for CCL2/CCR2 axis in monocytes, myeloid and plasmacytoid cells recruitment toward *MYCN*-nonamplified neuroblastoma, and suggest that the recruited myeloid DCs will further recruit T lymphocytes by means of CCL19 and CCL22. We also show that *MYCN*-nonamplified tumors present a very unusual microenvironment that can favor DC responses. Further preclinical work will be needed to define drugs that could restore CCL2 expression by neuroblastoma tumors, to favor immune cells recruitment, and immunologic response of high-risk neuroblastoma.

## Supplementary Material

Figure S1 Supplementary figure 1 shows chemokine arrays details: row data for culture medium, map of the array, and normalized data for all chemokines

Figure S2 Supplementary figure 2 shows flow cytometry gating strategy for cell subsets identification (CCR2 phenotyping)

Figure S3 Supplementary figure 3 shows flow cytometry gating strategy and raw data in calcium mobilization experiments in all cell subsets

Figure S4 Supplementary figure 4 shows flow cytometry gating strategy for cell subsets identification (migration experiments)

Figure S5 Supplementary figure 5 shows analysis of MYCN level in NB tumors in GSE62564 according to MYCN amplification status

Figure S6 Supplementary figure 6 shows differential CCL2 level according to MYCN amplification status in GSE 3960, and correlations between CCL2 level and immune cell infiltrate

Figure S7 Supplementary figure 7 shows heat map and dot plots illustrating correlations between CD3E and chemokines levels, detailed migration of CD4 and CD8 lymphocytes driven by cell lines, correlations between CD3E and CD1c and between CCL19/CCL22 and APC specific markers.

Figure S8 Supplementary figure 8 shows HLA class I level of expression in NB cell lines, and heat map illustrating relations between chemokines, immune cell subsets and HLA class I associated genes levels.

Figure S9 Supplementary figure 9 shows IL-12 and IL-8 contents in supernatants after activation of PBMC in co-culture with NB cell lines.
