# Supplementary figures and images for: Impaired Antitumor Immune Response in MYCN-amplified Neuroblastoma Is Associated with Lack of CCL2 Secretion and Poor Dendritic Cell Recruitment
Source: Cancer Res Commun. 2022 Jul 5;2(7):577–89. doi: 10.1158/2767-9764.CRC-21-0134 (PMC10010397; doi:10.1158/2767-9764.CRC-21-0134)

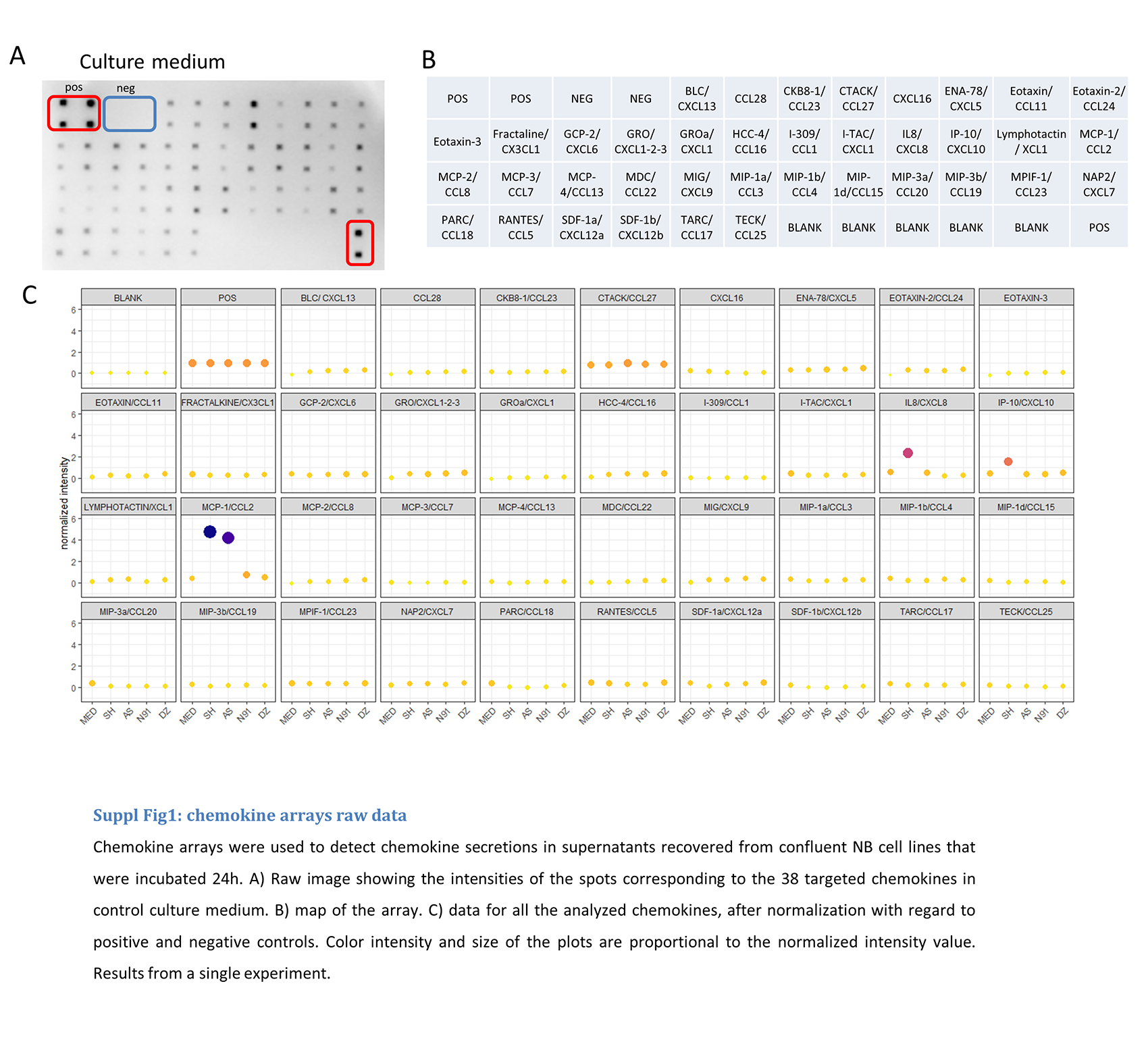

Supplement: Figure S1 — Supplementary figure 1 shows chemokine arrays details: row data for culture medium, map of the array, and normalized data for all chemokines [file crc-21-0134-s01.png]

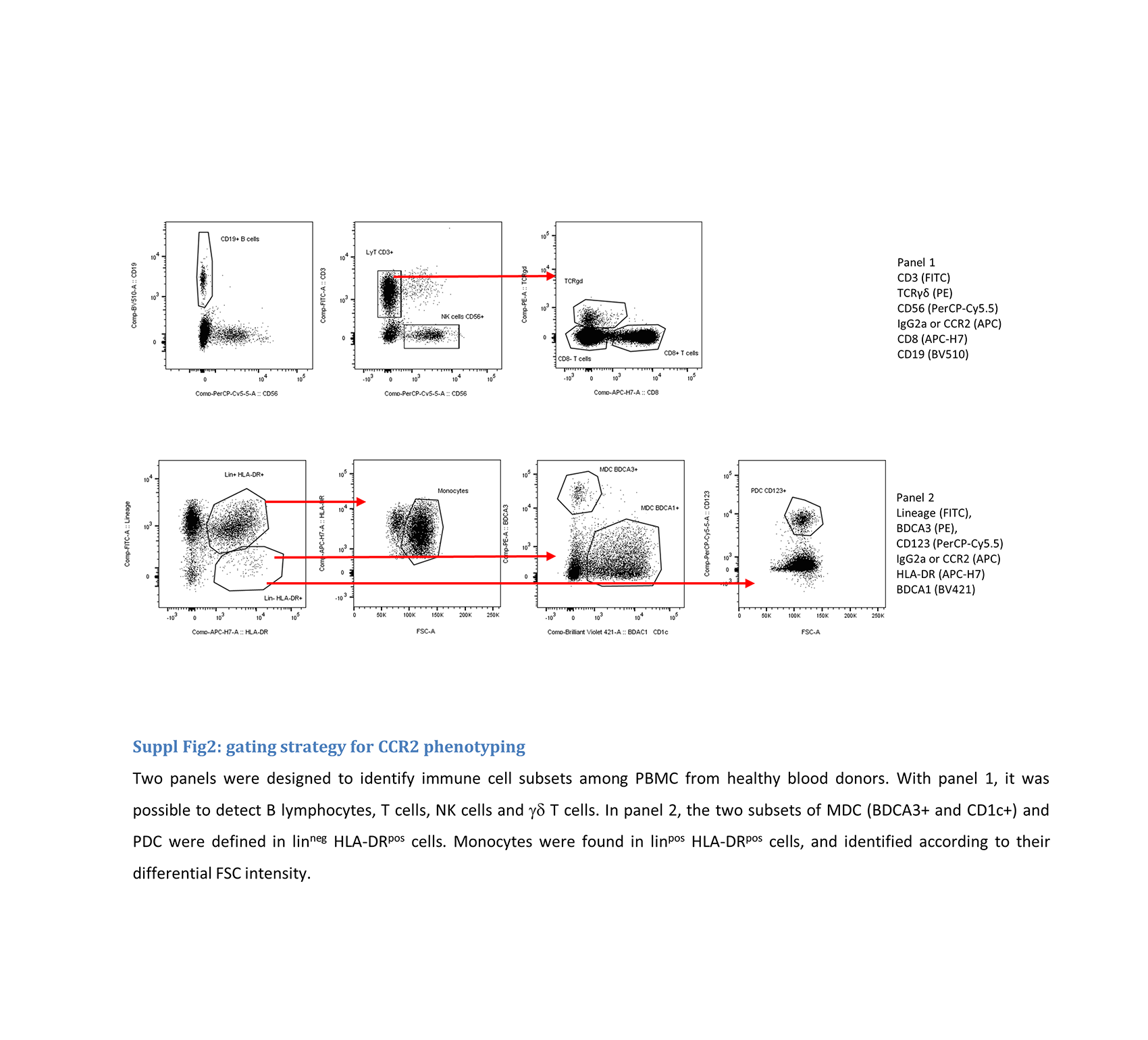

Supplement: Figure S2 — Supplementary figure 2 shows flow cytometry gating strategy for cell subsets identification (CCR2 phenotyping) [file crc-21-0134-s02.png]

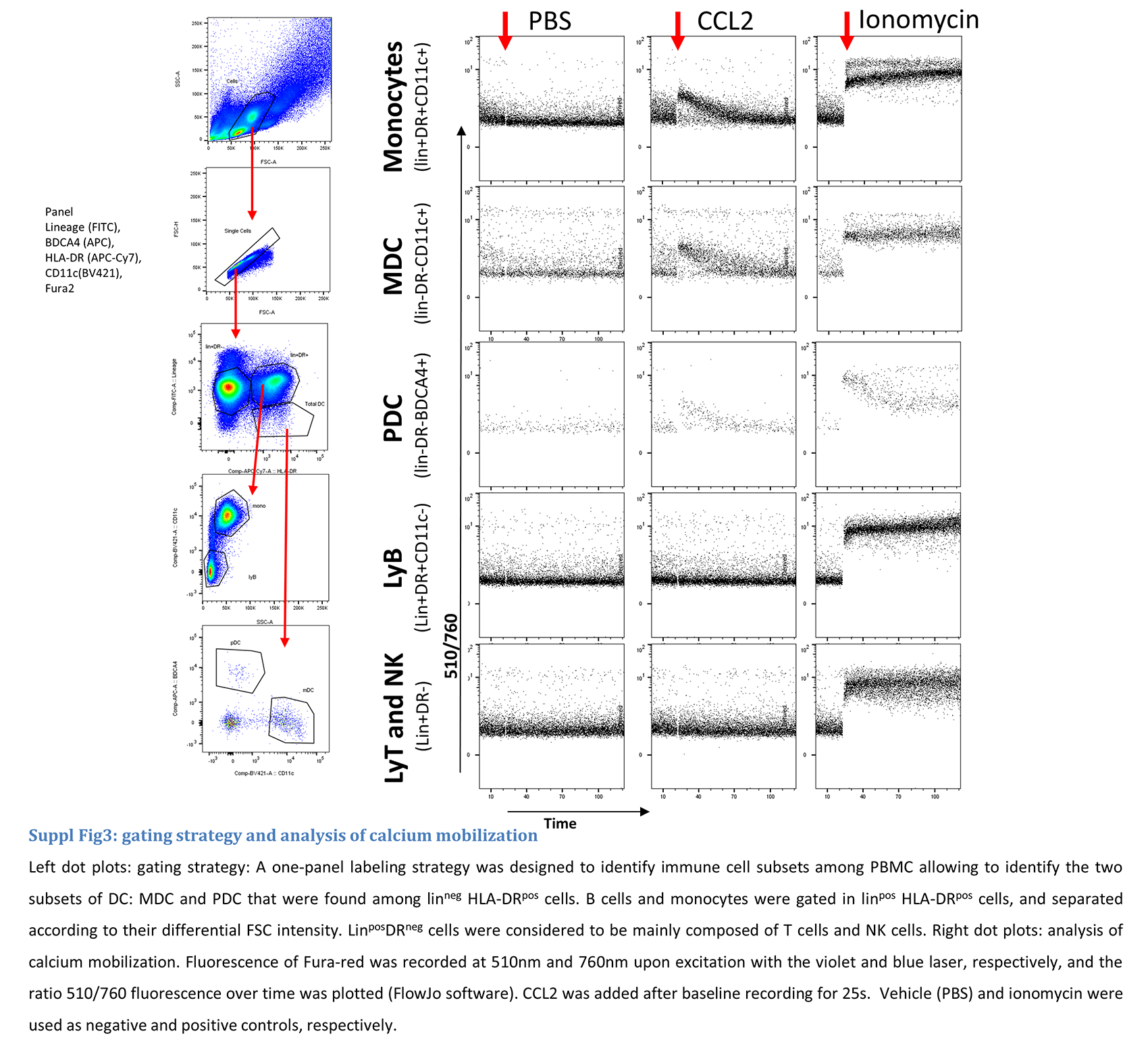

Supplement: Figure S3 — Supplementary figure 3 shows flow cytometry gating strategy and raw data in calcium mobilization experiments in all cell subsets [file crc-21-0134-s03.png]

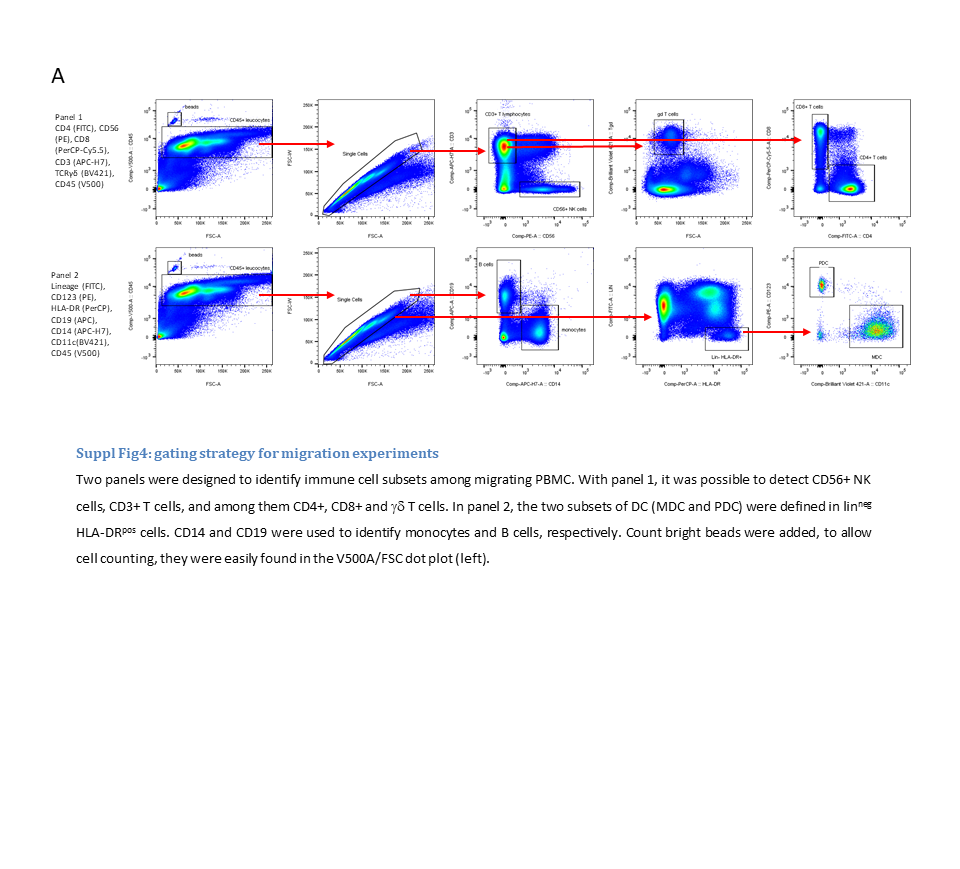

Supplement: Figure S4 — Supplementary figure 4 shows flow cytometry gating strategy for cell subsets identification (migration experiments) [file crc-21-0134-s04.png]

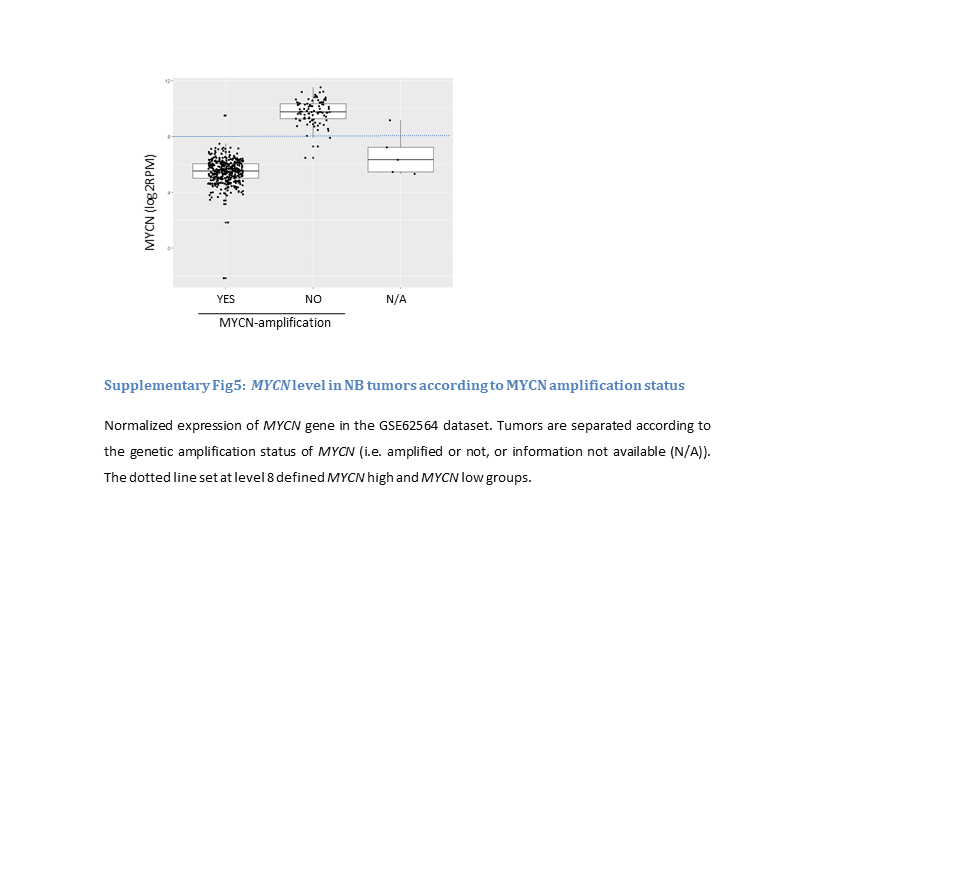

Supplement: Figure S5 — Supplementary figure 5 shows analysis of MYCN level in NB tumors in GSE62564 according to MYCN amplification status [file crc-21-0134-s05.png]

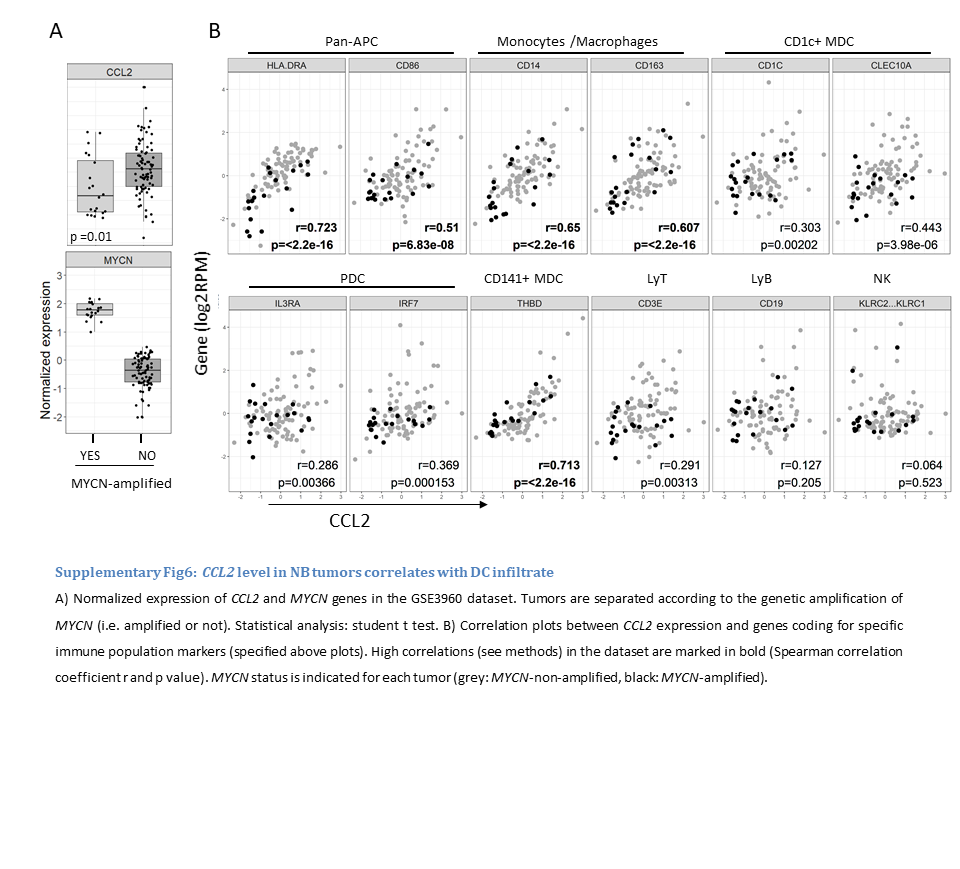

Supplement: Figure S6 — Supplementary figure 6 shows differential CCL2 level according to MYCN amplification status in GSE 3960, and correlations between CCL2 level and immune cell infiltrate [file crc-21-0134-s06.png]

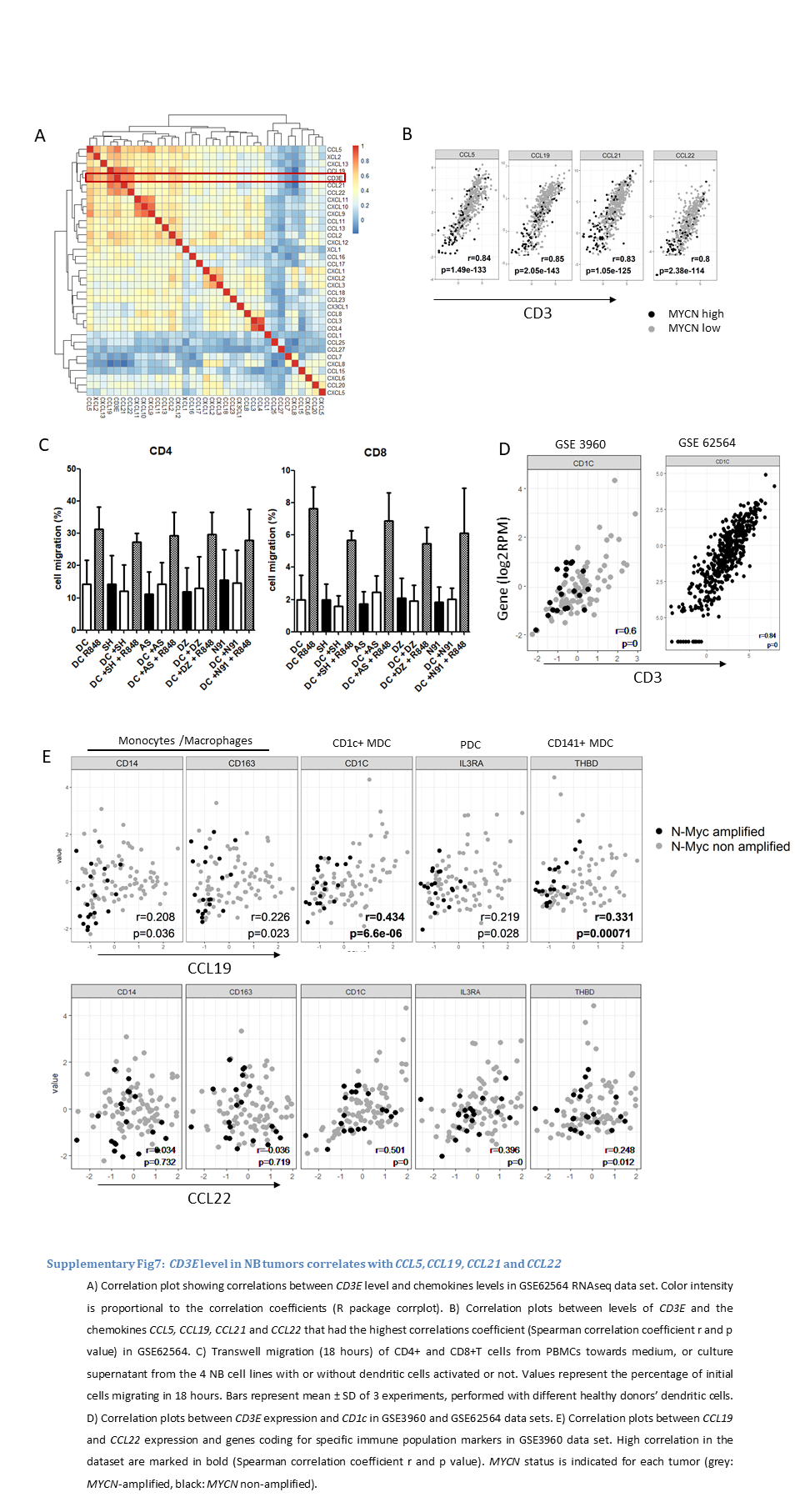

Supplement: Figure S7 — Supplementary figure 7 shows heat map and dot plots illustrating correlations between CD3E and chemokines levels, detailed migration of CD4 and CD8 lymphocytes driven by cell lines, correlations between CD3E and CD1c and between CCL19/CCL22 and APC specific markers. [file crc-21-0134-s07.png]

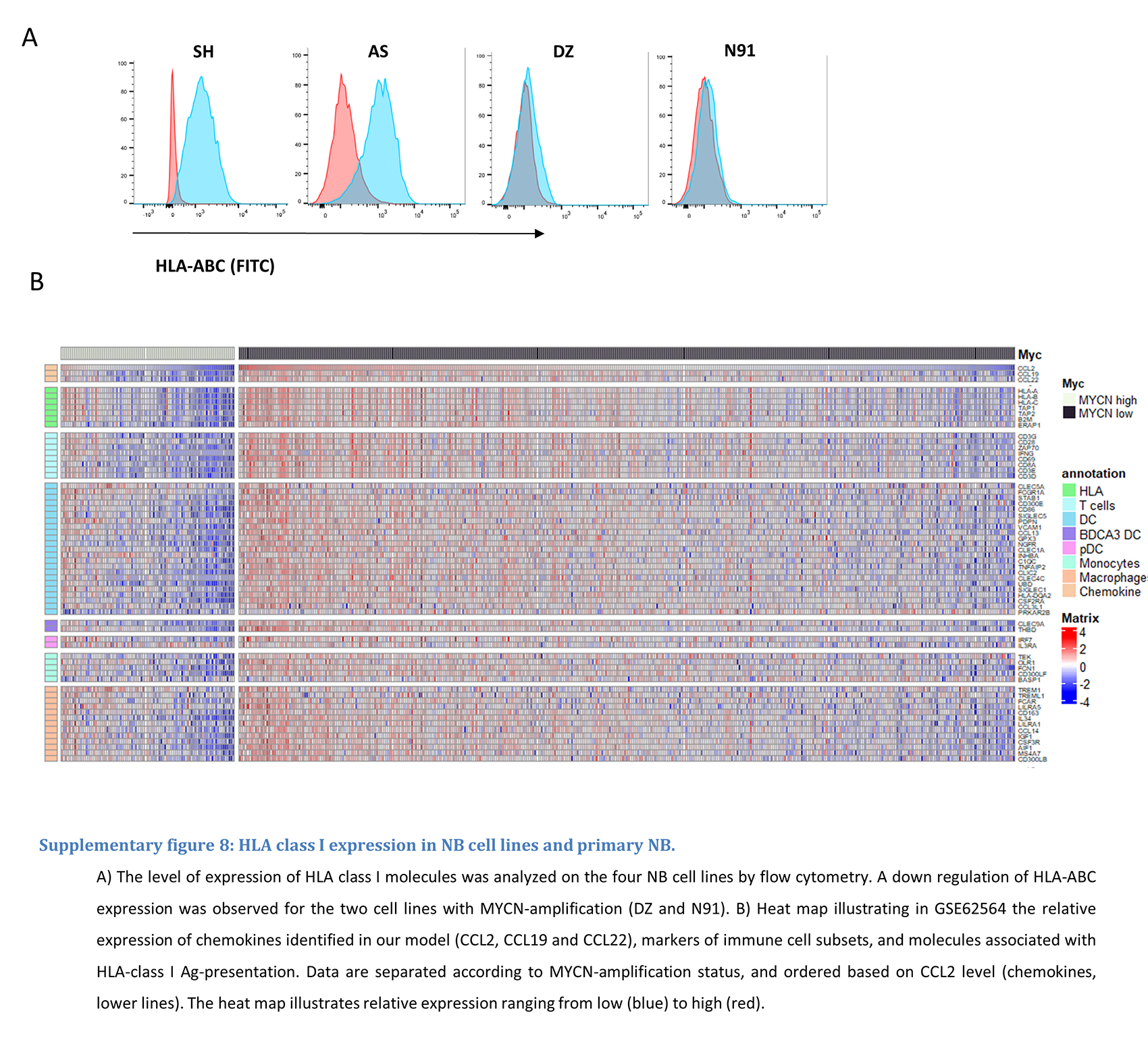

Supplement: Figure S8 — Supplementary figure 8 shows HLA class I level of expression in NB cell lines, and heat map illustrating relations between chemokines, immune cell subsets and HLA class I associated genes levels. [file crc-21-0134-s08.png]

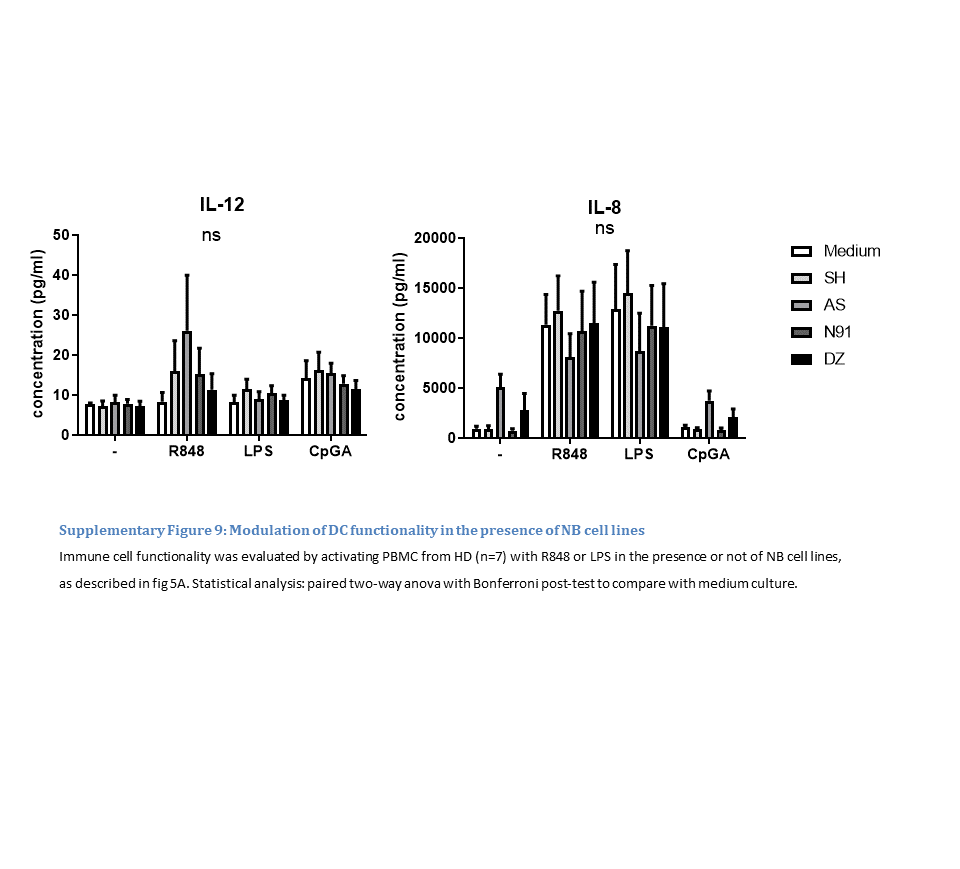

Supplement: Figure S9 — Supplementary figure 9 shows IL-12 and IL-8 contents in supernatants after activation of PBMC in co-culture with NB cell lines. [file crc-21-0134-s09.png]
